# Draft Genome Sequence of *Brucella abortus* BCB027, a Strain Isolated from a Domestic Deer

**DOI:** 10.1128/genomeA.00130-12

**Published:** 2013-01-31

**Authors:** Lulu Wang, Yefeng Qiu, Zeliang Chen, Jie Xu, Zhoujia Wang, Yuehua Ke, Tiefeng Li, Dali Wang, Liuyu Huang, Yaqin Yu, Qing Zhen

**Affiliations:** a Department of Epidemiology and Biostatistics, Key Laboratory of Zoonosis, Ministry of Education, School of Public Health, Jilin University, Changchun, Jilin, China; b Experimental Animal Center, Academy of Military Medical Science, Beijing, P. R. China; c Department of Infectious Disease Control, Beijing Institute of Disease Control and Prevention, Beijing, China; d Plague and Brucellosis Prevention and Control Base, Chinese Centers for Disease Control and Prevention, Baicheng, Jilin, P. R. China

## Abstract

Many *Brucella* species are isolated from nonpreferred hosts, and these bacteria may show genetic differences from isolates from the preferred hosts. Here, we report the draft genome sequence of *Brucella abortus* BCB027, a novel strain isolated from a domestic deer.

## GENOME ANNOUNCEMENT

Brucellosis is an infectious disease caused by bacteria of the genus *Brucella*, characterized by abortion and infertility in many mammal species. This disease is considered one of the most important zoonoses worldwide (1). Brucellosis is caused by the Gram-negative, facultative intracellular bacterium *Brucella*, which is classified as containing different species according to pathogenicity, host preference, and phenotypic characteristics. These classical *Brucella* species display a marked host range, for example, *B. melitensis* for sheep and goats, *B. abortus* for cattle, and *B. canis* for dogs (2). These bacteria constitute genetically committed groups that are evolutionarily linked to their preferred hosts and for which biologically meaningful species can be defined (3). On the other hand, the existence of nonunique hosts also indicates that each of these species has the capability to infect animal hosts other than its preferred species. Infection of a nonpreferred host means that the bacteria must adapt to the host (4). Therefore, the infection of a nonpreferred host might induce genetic polymorphic changes in the bacteria. Brucellosis is epidemic in China, and *Brucella* isolates have been isolated mainly from their preferred animal hosts (5). We also found that *Brucella* strains have been isolated from some nonpreferred hosts. Here, we report the genome sequence of *B. abortus* BCB027, a strain isolated from a domestic deer, an important economic animal in Jilin Province. Genome sequence comparison of this strain to that isolated from its natural host might provide information about its host adaptation and microevolution.

The genomic DNA of BCB027 was isolated from bacterial culture and sequenced with a HighSeq 2000 sequencer with paired-end protocol. After filtering of low-quality reads, the sequencing reads were assembled with the CLCBIO genomics workbench version 5.5 by the *de novo* assembly method. A total of 292 contigs were generated, 123 with a length of >10 kb and 281 with a length of >1 kb. The average length of the contigs was 11.09 kb and the total length was 3,240,915 bp. The final approximate coverage for these contigs was about 130×.

We then annotated the genome sequence. Open reading frames (ORFs) were predicted with RAST (6). rRNA was predicted by using RNAmmer (7), and tRNA was identified with tRNAscan-SE 1.21 (8). A total of 3,287 ORFs were predicted, including 3,240 protein-coding sequences, 43 tRNAs, one copy of 5S RNA, two copies of large-subunit rRNA, and one copy of small-subunit rRNA. The draft genome sequences were compared with virulent *B. abortus* 9-941. A total of 137 small deletions (34 in coding regions) and 3,507 polymorphism sites (2,731 in coding regions) were identified. This implied that strains that survived in nonpreferred hosts might induce large genetic changes in *Brucella* isolates. Further detailed analysis of these genetic polymorphisms will contribute to understanding of their roles in host adaptation and bacterial microevolution.

### Nucleotide sequence accession numbers. 

The draft genome sequences of *B. abortus* BCB027 are available in GenBank under accession number ALOL00000000. The versions described in this paper are the second versions: ALOL02000000.
